# The Unique Capability of Endolysin to Tackle Antibiotic Resistance: Cracking the Barrier

**DOI:** 10.3390/jox15010019

**Published:** 2025-01-25

**Authors:** Abdus Sabur, Angkan Khan, B. Borphukan, Abdur Razzak, M. Salimullah, Muslima Khatun

**Affiliations:** 1 Animal Biotechnology Division, National Institute of Biotechnology, Savar, Dhaka 1349, Bangladesh; abdussabur.btge@gmail.com; 2 Infectious Diseases Division, International Centre for Diarrheal Disease Research, Bangladesh, Mohakhali, Dhaka 1212, Bangladesh; muhammad.khan@icddrb.org; 3 Department of Crop and Soil Sciences, Washington State University, Pullman, WA 99163, USA; bhabesh.borphukan@wsu.edu; 4 Bioassay Department, Eurofins Biopharma, Columbia, MO 65201, USA; razzak.mdabdur@bpt.eurofinsus.com; 5 Molecular Biotechnology Division, National Institute of Biotechnology, Savar, Dhaka 1349, Bangladesh; salim2969@nib.gov.bd

**Keywords:** endolysins, bacteriophages, multidrug-resistant bacteria, protein engineering, phage therapy, holins, spanins, mutagenesis, synergism

## Abstract

The lack of new antibacterial medicines and the rapid rise in bacterial resistance to antibiotics pose a major threat to individuals and healthcare systems. Despite the availability of various antibiotics, bacterial resistance has emerged for almost every antibiotic discovered to date. The increasing prevalence of multidrug-resistant bacterial strains has rendered some infections nearly untreatable, posing severe challenges to health care. Thus, the development of alternatives to conventional antibiotics is critical for the treatment of both humans and food-producing animals. Endolysins, which are peptidoglycan hydrolases encoded by bacteriophages, represent a promising new class of antimicrobials. Preliminary research suggests that endolysins are more effective against Gram-positive bacteria than Gram-negative bacteria when administered exogenously, although they can still damage the cell wall of Gram-negative bacteria. Numerous endolysins have a modular domain structure that divides their binding and catalytic activity into distinct subunits, which helps maximize their bioengineering and potential drug development. Endolysins and endolysin-derived antimicrobials offer several advantages as antibiotic substitutes. They have a unique mechanism of action and efficacy against bacterial persisters (without requiring an active host metabolism); subsequently, they target both Gram-positive and Gram-negative bacteria (including antibiotic-resistant strains), and mycobacteria. Furthermore, there has been limited evidence of endolysin being resistant. Because these enzymes target highly conserved links, resistance may develop more slowly compared to traditional antibiotics. This review provides an overview and insight of the potential applications of endolysins as novel antimicrobials.

## 1. Introduction

One of the main public health concerns of the twenty-first century is antimicrobial resistance; however, accurate estimates of the net global health burden resulting from bacterial antibiotic resistance are lacking [1,2]. Numerous studies have estimated the burden of resistance to particular combinations of clinical disease, bacterial agents, antibiotics, and better healthcare facilities than contexts (mostly hospitals in industrialized countries). However, these estimates are only approximate due to significant knowledge gaps and reliance on extrapolating from small-scale studies.

The “burden” of infectious diseases is measured using a variety of metrics, such as length of hospital stay, duration of morbidity, disability-adjusted life years, and cost of care. Precisely defining the burden of antibiotic resistance is a crucial first step. We propose the most relevant definition as the total number of deaths linked to antibiotic therapy failure due to antibiotic resistance. This definition does not equate to the total number of deaths among patients with antibiotic-resistant infections and could be significantly lower for two main reasons: first, not all patients with potentially resistant infections receive treatment with clinically indicated antibiotics; second, for those who do, the measurable difference in outcome between patients with susceptible and resistant infections may be negligible. Formally, the number of deaths that would not occur if antibiotic resistance were eradicated is known as the population-attributable fraction (PAF), also known as the etiological fraction. PAF is rarely used to assess the global burden of resistance since such data are rarely accessible [2]. According to a 2015 assessment, by 2050, multidrug-resistant (MDR) infections are expected to cause an additional 10 million deaths worldwide [3].

## 2. Global Antibiotic Usage

According to recent estimates, around 70 billion doses of antibiotics are consumed annually worldwide [4]. The most common antibiotic classes, accounting for more than half (55%) of all permitted antibiotics, were fluoroquinolone (18%), tetracycline (17%), penicillin (10%), and sulfonamide (10%) [5]. The World Health Organization (WHO) last released generic guidelines for the therapeutic use of antibiotics in 2023. These guidelines, along with more recent national and international recommendations, emphasize the importance of considering local circumstances—particularly local patterns of antibiotic resistance—but are not prescriptive. Consequently, usage patterns can differ greatly between places [6].

Hospital-level data on antibiotic consumption are available in some countries, but again, these data are not consistently connected to the ailments [7]. Current antibiotic resistance patterns influence antibiotic usage. For example, due to resistance, significant Gram-negative bacterial infections may not be treated with amino-penicillins alone, and require combination therapies.

A recent survey by the World Health Organization (WHO) provided extensive information on antibiotic resistance worldwide [8]. Nonetheless, for the majority of bacterial species and antibiotic uses, less than half of the global population was represented, as many countries provided only limited data (testing 30 isolates). This survey highlighted significant differences in the types of isolates studied and, in the techniques used for resistance testing. This gap complicates the accurate determination of the PAF calculation, particularly the fraction of patients with bacterial infections resistant to the antibiotics used for treatment. This issue is compounded by the lack of data linking antibiotic usage to patient clinical states.

## 3. Causes of the Crisis in Antibiotic Resistance

### 3.1. Misuse of Antibiotics

In 1945, Sir Alexander Fleming drew attention to the risk of antibiotic misuse and overuse, a concern that remains relevant today [9,10]. The misuse of antibiotics significantly contributes to the emergence of resistance [10]. Epidemiological research links antibiotic use directly to the formation and spread of resistant bacterial strains [11]. Bacterial resistance genes can be inherited or acquired through horizontal gene transfer (HGT) via plasmids, spreading resistance among various bacterial species [12]. Similarly, natural selection pressures allow antibiotic-resistant bacteria [mutant] to proliferate [13].

**Figure 1:**
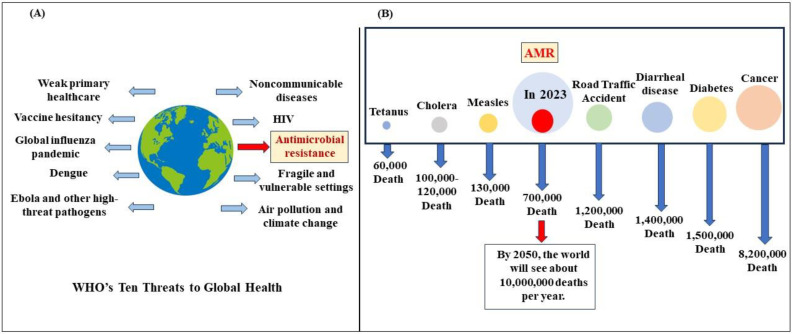
(**A**) WHO’s ten threats that burden human health with highlighted antibiotic resistance. (**B**) The number of antimicrobial-resistance-related deaths occurring annually compared to other leading causes of death.

Despite warnings, the global over-prescription of antibiotics continues to exacerbate resistance, indicating a need for strict control [14]. The antibiotic misuse is exacerbated due to the lack of regulations, and retail sold and over-the-counter sales [11,14]. Figure 1 depicts the threat caused by antibiotic resistance.

#### 3.1.1. Bad Prescription Practices

An improper prescription of antibiotics significantly encourages the growth of resistant bacteria [15]. Research indicates that 30% to 50% of instances involve inappropriate treatment indications, agent selections, or antibiotic medication durations [15,16]. Additionally, studies show that 30% to 60% of antibiotics administered in intensive care units (ICUs) are unnecessary, inappropriate, or suboptimal [16].

#### 3.1.2. Widespread Use in Agriculture

Antibiotic use in agriculture significantly impacts the environmental microbiome. Up to 90% of the antibiotics administered to livestock are excreted in feces and urine, leading to their widespread distribution by surface runoff, groundwater, and through natural fertilizers [10,15]. The use of antibiotics as insecticides also has significant geographic effects and may increase the ratio of resistant to susceptible bacteria in the environment [17]. Antibacterial products marketed for cleaning or hygiene may also prevent the development of immunity to environmental antigens, weakening immune system adaptability, and potentially increasing morbidity and mortality from typically non-virulent infections [14,17].

#### 3.1.3. Limited Supply of New Antibiotics

The pharmaceutical industry considers novel antibiotic development as financially imprudent [10]. Pharmaceutical companies prefer to invest in chronic disease treatments like diabetes, asthma, or gastric reflux due to their higher profitability compared to antibiotics because of their short-term use and considered curative treatment or care [10,17,18,19,20]. Additionally, antibiotics are relatively inexpensive. A course of newer antibiotics costs USD 1000 to USD 3000, compared to tens of thousands of dollars spent on chemotherapy [10,18,19,20]. A cost–benefit analysis by the Office of Health Economics in London found that a drug for a neuromuscular condition has an approximate USD 1 billion net present value (NPV), while a new antibiotic has only about USD 50 million [10].

#### 3.1.4. Regulating Obstacles

Regulatory approval is a significant barrier for companies discovering novel antibiotics [18,20]. Several issues have been identified as obstacles to obtaining regulatory approval, such as bureaucracy, ambiguity, differences in national requirements for clinical trials, changes in licensing and regulatory policies, and inefficient channels of communication [20]. The U.S. Food and Drug Administration (FDA) has altered clinical trial guidelines, making antibiotic studies particularly challenging [19]. Trials comparing antibiotics with placebos are unethical, so studies must show new treatments are not inferior to existing ones, often requiring large sample sizes and high costs [19,20]. This complexity makes antibiotic development unprofitable and unattractive. Although small businesses have stepped into phase 3, clinical trials remain prohibitively complex and expensive [20].

## 4. What Is the Solution?

The use of bacteriophages and the endolysins produced by them, such as new antimicrobials, is one strategy that shows promise. At the end of the phage’s lytic cycle, these proteins naturally degrade the peptidoglycan (PG) of the bacterial host cell. This action causes rapid osmotic lysis of the host, leading to cell death and the release of progeny phages [21].

## 5. Bacteriophages

Bacteriophages, or phages, are viruses that specifically infect bacteria [22]. Phages have co-evolved with their bacterial hosts, maximizing their ability to proliferate within the host cell and their method of external release. Double-stranded DNA phages express virion-associated peptidoglycan hydrolase (VAPGH) proteins, which attach to cell surface antigens with high specificity and degrade the bacterial cell wall, allowing the phage to inject its DNA into the host cell [23,24]. For nearly a century, these bacteriophages have been used to treat bacterial infections [25].

### 5.1. Phage Therapy

The first clinical research with phages was conducted in 1921, involving direct phage application to six patients who have *Staphylococcal* boils [26]. Numerous clinical phage experiments against a range of pathogens, such as *Salmonella typhimurium*, *E. coli*, *Klebsiella pneumoniae*, *Staphylococcus aureus*, and *Pseudomonas aeruginosa*, have been reported so far. There are numerous phage-therapy-related businesses in different nations that produce commercial products [27]. As a result, phage therapy presents new perspectives and methods for the effective bio-controlling of a variety of antibiotic-resistant bacteria without causing any negative effects on humans. The rising prevalence of antibiotic-resistant bacteria over the past 20 years has renewed the interest in using phages and phage-derived proteins to combat “superbugs” [28,29].

### 5.2. How About Using Phages Alone?

Endolysins have shown excellent benefits in experimental settings; thus, one may wonder why a clinician would not just use the parental phage. It may seem inefficient to clone the endolysin protein and use it in a recombinant expression system when it is naturally present in the phage. However, endolysins provide several advantages over phages, despite the challenges associated with genetic modifications.

## 6. Endolysins

**Figure 2:**
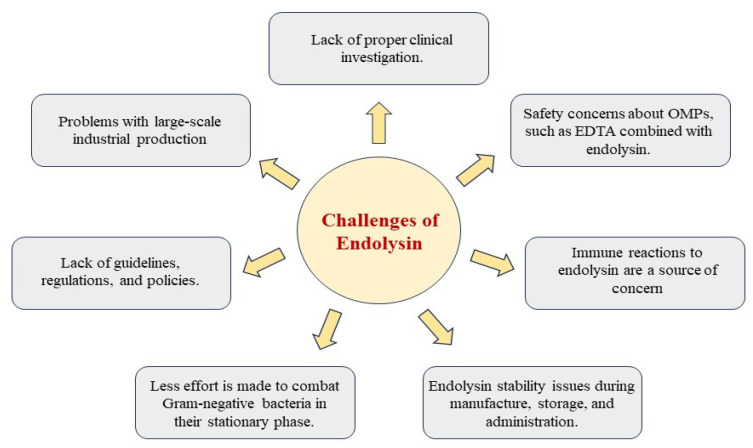
Challenges of endolysins.

Peptidoglycan hydrolases encoded by phages are called endolysins. These enzymes and a related holin protein accumulate inside the host cell without the virion’s assistance. Holins create pores in the cytoplasmic membrane that help endolysins access the bacterial peptidoglycan, as endolysins lack their signal sequences [4]. This coordinated action of holins and endolysins is necessary for the successful lysis of a bacterial cell. Recently, endolysins have drawn attention as potential antimicrobials due to their exogenous lytic actions. Figure 2 illustrates the challenges of endolysins

Endolysins are particularly effective against Gram-positive bacteria which lack an outer membrane for protection [29]. While their use against Gram-negative bacteria is more challenging due to the outer membrane, it is not entirely precluded.

### 6.1. Endolysins and Associated Phage Proteins

**Figure 3:**
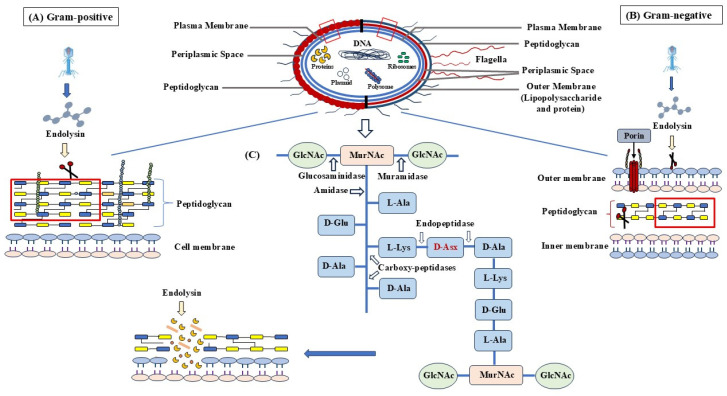
Endolysin-mediated bacterial cell wall degradation mechanism. (**A**) The exposed peptidoglycan layer in Gram-positive bacteria is effectively broken down by endolysins, which causes rapid cell lysis. (**B**) In Gram-negative bacteria, the outer membrane functions as a barrier, decreasing the efficacy of endolysin breakdown by preventing direct access to the peptidoglycan. (**C**) A detailed illustration of endolysin activity, highlighting specific enzymatic cleavage sites within the bacterial cell wall, ultimately leading to its breakdown. This figure has been inspired by [30].

The main phage-encoded proteins associated with the function of lysins are holins, signal peptides, and spanins [21]. Main barrier of gram negative bacteria that prevents endolysins has been highlighted in Figure 3.

Holins

Holins are membrane proteins that assist the transport of lysins across the cytoplasmic membrane and break down the peptidoglycan [21]. These proteins accumulate in the host bacteria’s cytoplasmic membrane, causing lesions that allow lysins to access the peptidoglycan [21]. Holins are classified into one of three groups according to the number of transmembrane domains (TMDs) they possess, which is determined by their membrane topology [21].

Signal Sequences

Reports have indicated the presence of a signal sequence in the N-terminal region of lysins [31,32]. Some endolysins posses signal sequences; Sao-Jose et al. provided initial experimental proof of secretory lysins, showing that the expression of *Pneumococcal* lysin Lys4 produced precursor and mature enzyme forms [31]. Furthermore, supporting data for the *Lactobacillus fermentum* phage lysin Lyb5 was reported. A chimeric linkage between the N-terminal of lysin and the nucB gene from *S. aureus* resulted in the export of NucB protein into the surrounding environment after gene expression in *L. lactis*. Additionally, 20 min after induction, the normally rod-shaped *E. coli* assumed a spherical shape due to the production of Lyb5 secretory lysin. Thus, it was proposed that lysin export to the cell wall was the cause of the morphological alteration [33].

Spanins

A third class of lysis proteins, known as spanins, has also been discovered [34]. These proteins comprise an outer membrane lipoprotein that integrates into the inner membrane and has a C-terminal transmembrane domain [34]. The most well-characterized spanins are the lambda Rz and Rz1 proteins [34,35]. Recent research revealed that the spanin complex of the lambda phage is necessary for the lysis of bacterial cells; lysogens expressing lambda 7oling and endolysin genes, and, importantly, spanin-null mutants did not result in cell lysis but produced delicate spherical cells. This suggests that spanins play a crucial role in outer membrane rupture, controlled by the condition of the peptidoglycan layer [35].

### 6.2. Classification of Phage Lysins

**Figure 4:**
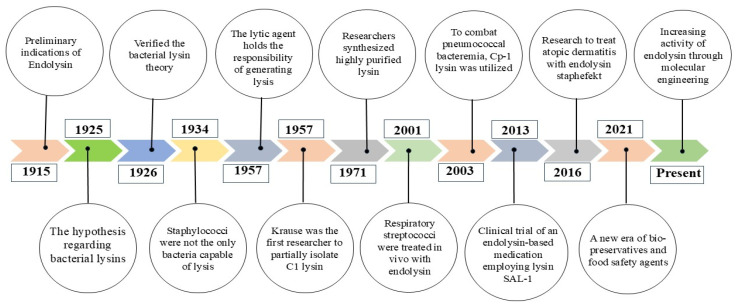
A timeline that provides an overview of the research effort on endolysin. After so many efforts from the dedicated researchers, it has come to this present situation and soon, the only goal will be to improve its efficacy and acceptance [40,41,42,43,44,45,46,47,48,49]. This figure has been inspired by Abdelrahman et al., 2021 [50].

Endolysins are typically classified based on their cleavage sites, including L-alanoyl-D-glutamate endopeptidases, N-acetylmuramoyl-L-alanine amidases, glycosidases (N-acetyl-D-glucosamidases), and lysozymes (N-acetylmuramidases) [36,37,38]. Endolysins typically contain one of four N-terminals and a cell wall-binding domain. Figure 4 describes the sequential improvements of endolysins.

Lysozymes: (N-acetylmuramidases) eliminate microorganisms through targeted hydrolysis. Peptidoglycan polymers are linked by ~−1, 4 glycosidic bonds between NAG and NAM monomers. They catalyze the degradation of the peptidoglycan polymers linked by hydrolyzing its bonds. This causes an imbalance in turgor pressure, resulting in bacterial lysis [39].

Glycosidases: (N-acetyl-\-D-glucosamidases) catalyze the hydrolysis of glycosidic linkages [39].

N-acetylmuramoyl-L-alanine amidases: also known as peptidoglycan amidases, hydrolyze the amide link that separates the glycan strand from the stem peptide between N-acetylmuramic acid and L-alanine residues [39].

L-alanoyl-D-glutamate endopeptidases and interpeptide bridge-specific endopeptidases: target peptides containing L-lysine and D-alanine–D-glutamate endopeptidases and interpeptide bridge-specific endopeptidases [39].

Different endolysins are derived from different genes and sources, or have different characteristics. Table 1 provides a clear view.

**Table 1:** Comparison of the selected phage endolysins.

Protein Name	Length	Gene	Source Organism	Antibacterial Activity	Effective Against G+ or G- Bacteria	Test Done In Vitro/In Vivo	Animal Model	MDR (YES/NO)	ATCC strain or Clinical Isolate	Expression Vector	Expression Host	pH	Temperature	Reference
LysMR-5	495	lysMR-5	*S. aureus* phage MR-5	*S. aureus ATCC 43300* *(MRSA),* *S. aureus ATCC 33,591 (MRSA), S. aureus ATCC 25923* *(MSSA),* and *S. aureus ATCC 29,213 (MSSA)*	Gram-positive	In Vitro	No	YES	ATCC	pET28a	*E. coli* BL21	7	37 °C	[51]
LYS_BPS13	91	E	Enterobacteria phage S13 (Bacteriophage S13)	*B. cereus*	Gram-positive	In Vitro	No	Not available	ATCC	pET15b	*E. coli* BL21	9.5	42–45 °C	[52]
LysB4	262	lysB4	Bacillus phage B4	*B. cereus*	Both	In vitro	No	Not available	ATCC	pET15b	*E. coli* BL21	8.0–10.0	50 °C	[53]
PlyB	326	plyB	Aspergillus nidulans	*Bacillus*	Not available	Both	Mouse	Not available	ATCC	pBAD24 (60)	*Escherichia coli* strain TOP10	Not available	Not available	[54]
PlyC	465	orf11	Streptococcus phage C1	*Staphylococcus aureus (MRSA), Enterococcus, E. coli, and Gram positive Lactococcus lactis*	Both	In vitro	No	Not available	Clinical Isolate	pEX and pET-22b	*E. coli* strain BL21 (DE3)	Not available	5 to 60 °C	[55]
Lys68	162	Lys68	Salmonella phage phi68	*Salmonella, Klebsiella, Pseudomonas* etc.	Gram-positive	In Vitro	No	Not available	Not available	pET-28a	*E. coli* BL21(DE3)	7	4 C to 40 °C	[56]
LysH5	481	LysH5	Staphylococcus phage phiH5 (Bacteriophage phiH5)	*Staphylococcus aureus and Staphylococcus epidermidis*	Gram-positive	In Vitro	No	Not available	Clinical Isolate	No	No	7	37 °C	[57]
Endolysin	185	ABgp46	Acinetobacter phage vB_AbaP_CEB1	*A. baumannii, S. typhimurium LT2, E.coli,* etc.	Both	In Vitro	No	Not available	Both	pET15b-ABgp46	*Escherichia coli* BL21(DE3)	4.0–10.0	Up to 50 °C	[58]
Putative phage lysin	245	phi7917_002	Streptococcus phage phi7917	*E. coli, Salmonella, B. subtilis, S. aureus, S. suis*	Both	Both	Mice	Not available	Both	pSJ2	*E. coli* BL21 (DE3)	6.0–9.0	Up to 50 °C	[59]
Ribonucleoside-diphosphate reductase, 1.17.4.1	695	PBC4_057	Bacillus phage PBC4	*B. cereus*	Gram-positive	In Vitro	No	Not available	ATCC	Not available	Not available	Not available	Not available	[60]
CHAP domain protein, Lysin	238 AA	VD13_036, X878_0033	Enterococcus phage VD13	*E. faecalis, Staphylococcus aureus, Escherichia coli DH5* *α*	Both	In vitro	No	YES	Both	pET21a	*E. coli* BL21(DE3)	4–10 (At 5 highest activity	4–100 (At 50 highest activity	[61]
ST01 protein	96	st01	*Escherichia coli*	*P. aeruginosa, K. pneumoniae, E. coli*	Gram negative	Both	Galleria mellonella larvae	YES	ATCC, KCTC, CCARM	pAS008 or pAS047	BL21 (DE3)	Not available	Not available	[62]
ClyC/NocO	434	clyC/nocO	Nodularia sp. LEGE 06071	*S. aureus, Enterococcus faecalis, Bacillus cereus*	Gram positive	Both	Mouse	YES	Both	pET28a	*E. coli* BL21(DE3)	Not available	4−65 °C	[63]
lysozyme	274	phiCTP1_gp29	Clostridium phage phiCTP1	*Clostridium species, lactic acid bacteria, Bacillus cereus.*	Gram positive	In vitro	None	YES	NCIMB (Aberdeen, UK), ATCC (Manassas, VA, USA), CECT (Valencia, Spain), the BCCM/LMG (Ghent, Belgium)	pET15b	*E. coli* BL21 (DE3)	Not available	Not available	[64]
N-acetylmuramoyl-L-alanine amidase	289	PHIM1EF22_0110	Enterococcus phage phiM1EF22	*E. faecalis*	Gram positive	In vitro	None	Not available	Both	pDP2	*E. coli* CG61	4–10 pH	10–60 °C	[65]
N-acetylmuramoyl-L-alanine amidase	233	PlyG, GAMMALSU_0017, GAMMAUSAM_0017	Bacillus phage Gamma	*Bacillus anthracis*	Gram positive	In vitro	None	YES	ATCC	pET-19b	*Escherichia coli* [BL21(DE3)	7	40	[66]
Portal protein	602 AA	ORF17	*Helicobacter pylori* bacteriophage KHP30	*H. pylori*	Gram negative	In vitro	None	YES	ATCC	Not available	*E. coli* BL21(DE3)	5–10 pH	10–55 °C	[67]
L-alanyl-D-glutamate peptidase	137	lys	Escherichia phage T5 (Enterobacteria phage T5)	*Escherichia coli*	Gram negative	In vitro	None	YES	National Collection of Micro-organisms IBPM RAS	pT5lys	*Escherichia coli* BL21(DE3)	3–10 pH	10–60 °C	[68]
Endolysin	133	elyY	*Yersinia enterocolitica* (type O:9)	*Yersinia enterocolitica, E. coli*	Gram negative	In vitro	None	YES	Both	pET28-elyY	*E. coli* BL21(DE3)	7	37 °C	[69]
N-acetylmuramoyl- L-alanine amidase	289	Thymidylate synthase	Enterococcus phage PBEF129	*E. faecalis*	Gram positive	In vitro	None	YES	Culture Collection of Antibiotic-Resistant Microorganisms in Korea	pET21-a(+)	*Escherichia coli* BL21 (DE3)pLyss	pH 5–9	37 °C	[70]
dihydrofolate reductase, 1.5.1.3	169	qdvp001_068	Vibrio phage qdvp001	*Vibrio parahaemolyticus*	Gram negative	In vitro	None	YES	ATCC	pET-30a	*E. coli* BL21	8	40 °C	[71]
Lysozyme, 3.2.1.17, CP-1 lysin, Endolysin, Muramidase	339	CPL1, 22	Streptococcus phage Cp-1 (Bacteriophage Cp-1)	*Streptococcus pneumoniae*	Gram positive	In vitro	None	YES	ATCC	pT7–7	*E. coli* BL21 (DE3)	8	37 °C	[72]
PHIKZ144	260	Transglycosylase gp144	Pseudomonas phage phiKZ	*Escherichia coli*	Gram negative	In vitro	None		Not available	pQE-30	*Escherichia coli*	7	40 °C	[73]
N-acetyl muramyl-L-alanine amidase	308	PlyPSA	Listeria phage PSU-VKH-LP041	*L. monocytogenes*	Gram positive	In vitro	None	YES	Not available	pASK-IBA5	*E. coli* K-12	7	45 °C	[74]
L-alanyl-D-glutamate peptidase	289	ply, ply500	Listeria phage A500 (Bacteriophage A500)	*Listeria species*	Gram positive	In vitro	None	Not available	ATCC	pASK-IBA5	*E. coli* K-12	7	45 °C	[75]
Endolysin	266	vB_BceM_AP3_0015	Burkholderia phage AP3	*E. coli, K. pneumoniae, P. aeruginosa, B. cenocepacia, S. enterica, Staphylococcus aureus and S. epidermidis*	Both	In vitro	None	YES	ATCC	pEXP5-CT/TOPO	*Escherichia coli* BL21-AI	pH 3–10	10–55 °C	[76]
Phage protein	68	SPN1S_0005	Salmonella phage SPN1S	*Salmonella typhimurium, Escherichia coli*	Gram-negative	In vitro	None	YES	ATCC	pET-28a	*E. coli* BL21 (DE3)	pH 4–10	40 °C	[77]

## 7. Applications of Endolysins

### 7.1. Application of Endolysins as Human Therapeutics

The decline in the effectiveness has made numerous infections potentially fatal, prompting research into phage-derived endolysins for treating human systemic and topical infections [78,79]. *Staphylococcus aureus* (*S. aureus*), a Gram-positive pathogen, can cause serious topical skin and nasal infections [80]. The rise inmethicillin-resistant and multidrug-resistant *S. aureus* (MRSA) has reduced the availability of effective treatments, making recombinant endolysins an essential option for managing *S. aureus* superbugs in clinical settings. A prevalent opportunistic pathogen that is present in the nasal mucosa of 20–40% of people, Staphylococcus aureus is a major contributor to the spread of infections acquired in hospitals and the community [81].

Gram-negative bacteria, such as *Acinetobacter baumannii* and *Pseudomonas aeruginosa*, are significant opportunistic pathogens in burn wounds [82]. Artilysins, an engineered endolysin, was found to be effective against these drug-resistant infections. Briers et al. (2014) successfully examined the action of novel endolysin LoGT-008 against *P. aeruginosa* and *A. baumannii* in a human neonatal keratin epidermal cell line model [83]. This new system using phage-derived lysins presents strong evidence to reduce antibiotic resistance compared to traditional phage cocktail therapy. Applications of endolysins as human therapeutics have been showed in Table 2.

### 7.2. Application of Endolysins in the Veterinary Sector

It has been suggested that endolysins are effective agents to combat most diseases associated with farm animals, including *Salmonella* species, *Clostridium perfringens*, *Streptococcus suis*, and *Paenibacillus larvae* [84,85]. Endolysins could be a way to combat *C. perfringens*, a Gram-positive multidrug-resistant pathogen that causes significant problems in poultry and can infect up to 95% of hens [86,87]. Anthrax, a serious zoonotic disease, has been targeted with PlyG endolysin from a gamma phage, showing therapeutic potential against *Bacillus anthracis* [88]. Similarly, clinical trials demonstrate that P128 hydrogel is effective against methicillin-resistant *Staphylococcus pseudointermedius* (MRSP). Table 2 have clarified these.

### 7.3. Endolysins in Food and Other Sectors

Food animals like pigs, cattle, and chickens, along with their products, can harbor drug-resistant infections [79]. Endolysin exhibits significant lytic activity against antibiotic-resistant gram positive and gram negative bacteria, inhibiting resistance [89]. Studies show that adding LysZ5 to soy milk effectively sterilizes it, preventing *Listeria monocytogenes* contamination [90]. Hydrostatic pressure combined with phage endolysins PlyP825, PlyP40, and Ply511 effectively treat *L. monocytogenes* [91].

Similarly, other endolysins are also found to effective against *Streptococcus equi* compared to other disinfectants, can sterilize 10^8^ CFU/mL of *S. equi* culture in 30 min at a 1 μg concentration [92]. Endolysins have also been explored as antibacterial agents to control lactic acid bacterial (LAB) contaminations in fuel ethanol fermentation [93]. Table 2 provides a clear view.

**Table 2:** This table shows the different applications of endolysins in human, veterinary treatments, food, and other sectors.

Application of Endolysins as Human Therapeutics				
Infection	**Species**	Antibiotics Resistance	Endolysin	Reference
Skin and respiratory infections	*Staphylococcus aureus* (MRSA)	Methicillin	LysK ClyS CF-301 MR-10 Staphefekt	[94,95,96,97,98,99,100,101]
Corneal infections	*Staphylococcus simulans*	Doxycycline, tetracycline	MV-L	[102,103]
Endocarditis, sepsis	*Staphylococcus epidermidis*	Rifamycin, fluoroquinolones, gentamicin, tetracycline, clindamycin	MV-L	[102,104]
Urinary tract infections, hemolytic–uremic syndrome, neonatal meningitis, hemorrhagic colitis	*Escherichia coli*	Penicillin, cephalosporins, cephamycins, carbapenems	MV-L	[102,105,106,107,108]
Nosocomial infections	*Enterococcus* *faecalis*	Vancomycin	PlyV12 EFAP-1, EFAL-1 IME-EF1 EF-P10 EC300 Lys170 LysEF-P10	[109,110,111,112,113,114,115,116]
Strep throat, pneumonia, skin infections, and meningitis	*S. pneumoniae*	Penicillin, erythromycin, clarithromycin, ceftriaxone	Cpl-1	[47,117,118,119,120,121]
Hospital-acquired pneumonia, community-acquired pneumonia, Community-acquired pneumonia, Bloodstream infections	*Acinetobacter* *baumannii*	Cephalosporin, carbapenem, ceftazidime, liprofloxacin	LysAB2 PlyF307	[122,123,124,125,126,127,128,129,130]
Malignant external otitis, endophthalmitis, endocarditis, meningitis, pneumonia, and septicemia	*P. aeruginosa*	Carbapenem, aminoglycosides (gentamicin, tobramycin, amikacin, neomycin, plazomicin, streptomycin)	OBPgp279	[126,130,131]
Recurrent urinary tract infections (rUTI), pneumonia, and bloodstream infections	*Klebsiella pneumoniae*	Carbapenem	LysPA26	[79,132,133]
Application of Endolysins in the Veterinary Sector				
Necrotic enteritis and sub-clinical disease	*Clostridium perfringens*	Tetracycline, bacitracin	CP25L Psm	[134,135,136,137,138,139,140]
Anthrax	*Bacillus anthracis*	Streptomycin	PlyG	[141,142,143,144]
Equine strangles	*Streptococcus equi.*	Vancomycin	PlyC	[145,146,147,148]
Arthritis, meningitis, septicemia, and endocarditis	*Streptococcus suis*	Penicillin, ampicillin	LySMP	[149,150,151,152]
Bloodstream infection intra-abdominal infection bacteremia endocarditis	*Enterococcus faecium* *E. faecalis*	Vancomycin, lincomycin, bambermycin, bacitracin, tetracycline, ciprofloxacin, erythromycin, kanamycin, penicillin, tylosin, streptomycin, vancomycin, gentamycin, streptogramins, avilamycin	PlyV12	[110,140]
Endolysins in Food and Other Sectors				
LysSA11	After 15 min of endolysin treatment, viable MRSA levels decreased in experimentally contaminated ham and pasteurized products.	*S. aureus*	Milk Products	[153,154]
Gp110	This endolysin, with a novel enzyme structure and N-acetylmuramidase lysis domain, exhibited exceptional in vitro activity against Salmonella and other Gram-negative pathogens.	*Salmonella* spp.	Sea Foods	[155,156]
LysCs4 SPN1S Lys68	Peptidoglycan from Gram-positive and Gram-negative bacteria from six distinct genera could be broken down by the refined lysozymes, which could also lyse *C. sakazakii* that had an outer membrane permeabilized.	*C. sakazakii*	Milk powders, herbal teas, and other dried products.	[56,157,158,159]
PlyBa Ply12 Ply21 LysBPS13 LysB4	Endolysins effectively combat 24 *B. cereus and B. thuringiensis* strains, contaminating food. Endopeptidase exhibits bactericidal activity against Gram-positive bacteria, including *B. cereus, B. subtilis*, and monocytogenes.	*B. cereus*	Dairy Products	[52,53,160,161]
CS74L CPT1l	It is also shown that these enzymes were active against *Clostridium acetobutylicum* and *C. tyrobutyricum* using the turbidity assay and fresh bacterial cells, indicating that they could be used as a potential bio preservative in cheese. Another endolysin that was recovered from a virulent phage was also described by the same family; however, this enzyme’s host range was more constrained.	*Clostridium sporogenes,* *Clostridium acetobutylicum,* *Clostridium tyrobutyricum*	In poultry, clostridial species are linked to food spoilage. Germinated *Clostridium sporogenes* and *Clostridium tyrobutyricum* have the potential to produce gases and acids in the dairy sector that alter the structural and sensory characteristics of cheeses.	[83,162,163]

## 8. Administration Routes

The successful administration of any therapeutic agent to the target infection site requires an appropriate administration route and delivery method that maintain the treatment’s stability and activity [164]. Currently, there are several ways to provide phage-derived enzymes such as transnasal, vaginal, and oral delivery methods; topical treatments (like creams, ointments, and gels); and injections (intravenous and intraperitoneal) [165,166]. Different administration routes have been showed in Table 3.

The oral administration of phage endolysins is challenging due to harsh gastric conditions. Encapsulation has been proposed to preserve enzymatic activity [166]. A preliminary human phase trial of SAL200 (recombinant version of phage endolysin SAL-1 derived from phage SAP-1) demonstrated the safety and efficacy of endolysin, with minor side effects like headaches and fatigue [167]. Pharmacokinetics and pharmacodynamics data following intravenous injection suggest that SAL200 effectively infects multiple *Staphylococci* species, including MRSA and vancomycin-resistant *S. aureus* (VRSA) [168,169].

**Table 3:** The selection of some endolysins and how they should be administered.

Target Pathogen	Phage	Enzyme (Endolysin)	Activity (Mode of Action)	Administration Route	References
MRSA	GH15	LysGH15	Amidase and endopeptidase	Intravenous and Intraperitoneal	[170,171]
*Streptococcus pneumoniae*	Cp1	Cpl-1	Muramidase	Intravenous, nasal, oral, aerosols, and Intraperitoneal	[120,172,173,174,175]
MRSA	MR11	MV-L	Amidase and endopeptidase	Intraperitoneal, nasal	[102]
*Streptococcus pyogenes*	MGAS5005 prophage	PlyPy	Endopeptidase	Intraperitoneal	[176]
MRSA	phiSH2 prophage, phiP68, phiWMY, phi80α, phi11 2854, prophage K	phiSH2, P68, LysWMY, 80αLyt2, phi11, 2638A, LysK	Amidase and endopeptidase	Intraperitoneal	[177]
*Pseudomonas aeruginosa*	phage PVP-SE1	Artilysin^®^ Engineered Endolysin-Based (PVP-SE1gp146)	Muramidase	Oral and topical	[165]
*Streptococcus agalactiae*	NCTC11261	PlyGBS	Endopeptidase and Muramidase	Intravaginal, oral and intranasal	[178]
*Pseudomonas aeruginosa*	*P. aeruginosa* phage	PlyPa03	Muramidase	Topical	[179]
*Streptococcus pneumoniae*	CP-7	Cpl-7	Muramidase	Immersion	[180]
*Acinetobacter baumannii*	RL-2015	PlyF307	Muramidase	intraperitoneal and Topical	[129]
*Enterococcus faecalis*	*E. faecalis* phage IME-EF1	LysIME-EF1	Endopeptidase	Intraperitoneal	[181]
*Acinetobacter baumannii*	SS3e	LysSS	Muramidase	Intraperitoneal	[182]
*Streptococcus pyogenes*	C1	PlyC	Amidase	Oral, nasal	[46]
*Bacillus* *anthracis*	γ-phage	PlyG	Amidase	Intraperitoneal	[100]

## 9. Functional Improvements

Various protein engineering techniques have been employed to enhance the activity and specificity of endolysins. These techniques include domain swapping and shuffling, endolysin mutagenesis, and other modifications leading to the active translocation of endolysins, and are comprehensively summarized in the following table. Table 4 could be very useful for improving the functional activity of endolysins.

**Table 4:** Potential approaches for molecular engineering and their potential applications.

Endolysin	Improvements	Assets	Activity Against	References
CHAPk	Full-length enzyme truncation	Enhanced solubility and catalytic activity	Methicillin-resistant *Staphylococcus aureus*	[183]
ClyS	Combination of EADs (enzymatically-active domain) and CWBDs (cell wall-binding domain) from several endolysins	Improved solubility and catalytic potential	Methicillin-resistant *S. aureus* (MRSA)	[97]
Art-Bp7e6	A random peptide was fused with the phage endolysin Bp7e	To create a chimeric endolysin library	ß-lactamase-resistant *E. coli*, *Salmonella enterica* serovar Enteritidis	[184]
EC300	Combination of CWBD of endolysin with virion-associated lysin	Enhanced effectiveness	Vancomycin-resistant *Enterococcus faecalis*	[65]
SA2-E-Lyso-SH3b, SA2-E-LysK-SH3b	Proteins with switched specificity are produced when distinct-origin CWBDs and EADs are combined	Enhanced catalytic efficiency and expanded lytic range	Cephalosporins-resistant *Listeria monocytogenes*	[147]
OBPgp279, PVP-SE1g-146	Combining endolysin and OMP (outer-membrane permeabilizer)	Improved capacity to combat Gram-negative bacteria.	Ceftazidime and tetracycline-resistant *Pseudomonas aeruginosa* and *Acinetobacter baumannii*	[165]
Art-175	AMP (antimicrobial peptide)-mediated endolysin fusion	Enhanced ability to combat Gram-negative bacteria	Methicillin-resistant *Staphylococcus aureus* (MRSA)	[165]
PlyG	Combination of EADs and CWBDs from several endolysins	Ability to manage the temperature	Clindamycin-resistant *C. perfringens*	[138]
LysAB2	Site-directed mutagenesis as well as truncation	Improvement of AMP	Colistin-resistant *A. baumannii*	[185]

### 9.1. Domain Swapping and Shuffling

The modular structure of lysins endows them with the potential for domain swapping and shuffling [138]. For example, a chimeric *Pneumococcal lysin*, created by linking the catalytic domain of one variant with the cell wall binding domain (CWBD) of another, showed increased bactericidal activity [186,187]. Conversely, a chimeric lysin from *Clostridium sporogenes* and *Clostridium difficile* domains had reduced lytic efficiency against *Clostridium tyrobutyricum* compared to the parent lysin [164]. Replacing the CWBD of *Clostridium perfringens* lysin with a thermophilic phage created a thermostable lysin [138]. Similarly, combining the CWBD of *Staphylococcal phage* lysin with the catalytic domain of *Enterococcal phage* resulted in an improved solubility of *Staphylococcal* phage lysin, along with broad lytic activity against *Staphylococci*, *Streptococci*, and *Enterococci* [188,189]. Domain shuffling can also affect lysin-binding properties. For instance, substituting the CWBD of *Listeria lysin* Ply118 with that of PlyPSA abolished lytic activity toward *Listeria serovar* 1/2 but enhanced activity toward serovar 4 [147]. This demonstrates the potential to create chimeric lysins with enhanced or specialized functions through domain swapping and shuffling, though outcomes vary depending on the domains combined.

### 9.2. Mutagenesis

Mutagenesis studies have also been employed to improve lysin activity. For example, substituting 15 amino acids in the CWBD of Pneumococcal phage lysin Cpl-7 enhanced its bactericidal activity and changed its net charge at neutral pH from −14.93 to +3 [190]. Conversely, deleting the CWBD has shown variable effects on lytic activity; in some cases, it improved lysis, while in others, it reduced or abolished activity. These effects are likely due to changes in the truncated lysin [190]. By employing mutagenesis, researchers can enhance or modify the activity of lysins, tailoring them to be more effective against specific bacterial targets.

### 9.3. Lysin Translocation

Protein engineering studies have focused on the active translocation of lysins across bacterial membranes. The signal peptide in the N-terminal region is vital for the translocation of lysin following expression [191]. Gaeng et al. demonstrated that attaching the *Lactobacillus brevis* S-layer protein’s signal peptide to *Listeria monocytogenes* phage lysin A511 enabled its active translocation within *Lactococcus lactis* host cells evident by creating an inhibition zone around the recombinant *L. lactis* in an agar medium with heat-inactivated *L. monocytogenes* [192]. A similar approach enabled the translocation of *Clostridium perfringens* lysin CP25L, which lysed *C. perfringens* cells in simulated gastrointestinal tract conditions without affecting other gut microflora [193].

Codon optimization is another promising avenue to enhance secretion efficiency, leading to a higher bactericidal activity of secreted lysin [176]. Rodríguez-Rubio and coworkers demonstrated increased activity of secreted lysin through codon optimization based on *L. lactis* codon usage [194]. By engineering lysins for active translocation, researchers can enhance their effectiveness in targeting and controlling specific bacterial pathogens, potentially leading to more effective antimicrobial therapies.

## 10. Synergism with Antibiotics

While endolysins have demonstrated efficacy as antimicrobials in numerous circumstances, lysins have also been employed in combination with other antimicrobial classes to achieve a synergistic impact against infection [97]. This synergy enhances therapeutic efficacy by significantly reducing the minimum inhibitory concentration (MIC) of antibiotics and the required dosage.

A study showed synergy between a chimeric lysin and a conventional antibiotic against methicillin-resistant *S. aureus* (MRSA). The chimeric lysin ClyS, combining the catalytic domain of phage lysin phiNM3 and the cell wall-binding domain of another *S. aureus* endolysin, was effective against various *S. aureus* strains. Combining ClyS with oxacillin in an MRSA septicemia model improved survival rates from 13% (control) to 80–82% (treated) [97]. Similarly, combined treatment with SAL200 and standard-of-care (SOC) antibiotics, including vancomycin and nafcillin, showed significant reductions in *S. aureus* concentration and antibiotic MIC in mouse and *Galleria mellonella* models [140]. SAL200 restored the sensitivity to nafcillin and vancomycin in strains approaching resistance, improving survival rates in infected models [176].

Letrado et al. demonstrated that Cpl-711 combined with antibiotics like amoxicillin, levofloxacin, vancomycin, and cefotaxime effectively treats multidrug-resistant *Streptococcus pneumoniae* strains. The combination showed strong synergistic associations, likely due to the antibiotic-induced degradation of the peptidoglycan cell wall, increasing vulnerability to endolysins. More recently, Kashani et al. used vancomycin in combination with the catalytic domains of endolysin LysK, CHAP, and amidase to treat MRSA, resulting in an eight-fold reduction in vancomycin MIC due to synergism [195].

## 11. Conclusions and Future Directions

With the global rise in multidrug-resistant bacterial infections, endolysins have drawn attention as a novel therapeutic strategy. Endolysins offer a promising alternative due to their lytic potential against various bacterial species in both human and veterinary medicine, as well as benefits in agriculture and biotechnology fields. Current research on multidrug resistance, safety, immunogenicity, and synergy with antibiotics has advanced the field of endolysins.

Endolysins are particularly effective against Gram-positive bacteria, but their activity against Gram-negative bacteria is limited by the outer membrane barrier. Nevertheless, they hold the potential to replace or enhance antibiotics in combating antimicrobial resistance. Engineering novel characteristics can enhance endolysins’ effectiveness equally against Gram-negative bacteria. As more endolysins are biochemically and structurally defined, our ability to design new enzymes improves, expanding our arsenal of lytic weapons. However, several hurdles must be overcome before this technology can be broadly utilized by practitioners and industries. While many researchers have isolated and characterized endolysin in vitro, determining their in vivo efficacy and operating parameters for human clinical use, food protection, animal husbandry, and environmental applications will be critical in the coming years. Additionally, the cost-effective scale-up of endolysin manufacturing is needed, as it is currently a major impediment to deployment.

In spite of having a lot of advantages, endolysins also have some disadvantages. Endolysins are more efficient against bacteria in the log-growth phase than bacteria in stationary phase; endolysins need to be stable during production, storage, and administration; endolysins have a short half-life in vivo because of the inflammatory response of cytokines and neutralizing antibodies; endolysins have not been adequately studied in clinical settings; there are no established norms and restrictions for endolysins; there are concerns about large-scale industrial manufacture of endolysins; and endolysins have been poorly studied in vivo.

To explore the potential of endolysins as a viable therapeutic alternative to antibiotics, future research should focus on several key areas. Firstly, comprehensive studies on the safety and efficacy of endolysins in human clinical trials are essential. Secondly, research should aim to understand the mechanisms of endolysins’ antibacterial action and their spectrum of activity against various pathogens. Investigating the development of resistance to endolysin and strategies to mitigate this risk will also be crucial. Thirdly, optimizing the delivery methods and formulations to enhance the stability and bioavailability of endolysins will be necessary for their successful application in clinical settings.

Novel approaches are required to overcome these immune reactions to endolysins, produce universal chimeric lysins, and penetrate the outer membrane of Gram-negative bacteria. Endolysins are showing promise as potential treatments, but further investigation is needed to evaluate how best to formulate and manufacture them for clinical trials. With continued research and technological advancements, endolysins could play a crucial role in combating antibiotic-resistant bacterial infections and improving public health outcomes.

## Data Availability

No new data were created or analyzed in this study.
